# Genetic diversity in *Capsicum baccatum* is significantly influenced by its ecogeographical distribution

**DOI:** 10.1186/1471-2156-13-68

**Published:** 2012-08-06

**Authors:** Elena Albrecht, Dapeng Zhang, Anne Deslattes Mays, Robert A Saftner, John R Stommel

**Affiliations:** 1 Keygene Inc., 155 Gibbs Street, Suite 405, Rockville, MD 20850, USA; 2 United States Department of Agriculture, Agriculture Research Service, Beltsville Agricultural Research Center, Plant Sciences Institute, Sustainable Perennial Crops Laboratory, Beltsville, MD 20705, USA; 3 United States Department of Agriculture, Agriculture Research Service, Beltsville Agricultural Research Center, Plant Sciences Institute, Food Quality Laboratory, Beltsville, MD 20705, USA; 4 United States Department of Agriculture, Agriculture Research Service, Beltsville Agricultural Research Center, Plant Sciences Institute, Genetic Improvement of Fruits and Vegetables Laboratory, Beltsville, MD 20705, USA

## Abstract

**Background:**

The exotic pepper species *Capsicum baccatum*, also known as the aji or Peruvian hot pepper, is comprised of wild and domesticated botanical forms. The species is a valuable source of new genes useful for improving fruit quality and disease resistance in *C. annuum* sweet bell and hot chile pepper. However, relatively little research has been conducted to characterize the species, thus limiting its utilization. The structure of genetic diversity in a plant germplasm collection is significantly influenced by its ecogeographical distribution. Together with DNA fingerprints derived from AFLP markers, we evaluated variation in fruit and plant morphology of plants collected across the species native range in South America and evaluated these characters in combination with the unique geography, climate and ecology at different sites where plants originated.

**Results:**

The present study mapped the ecogeographic distribution, analyzed the spatial genetic structure, and assessed the relationship between the spatial genetic pattern and the variation of morphological traits in a diverse *C. baccatum* germplasm collection spanning the species distribution. A combined diversity analysis was carried out on the USDA-ARS *C. baccatum* germplasm collection using data from GIS, morphological traits and AFLP markers. The results demonstrate that the *C. baccatum* collection covers wide geographic areas and is adapted to divergent ecological conditions in South America ranging from cool Andean highland to Amazonia rainforest. A high level of morphological diversity was evident in the collection, with fruit weight the leading variable. The fruit weight distribution pattern was compatible to AFLP-based clustering analysis for the collection. A significant spatial structure was observed in the *C. baccatum* gene pool. Division of the domesticated germplasm into two major regional groups (Western and Eastern) was further supported by the pattern of spatial population structure.

**Conclusions:**

The results reported improve our understanding of the combined effects of geography, ecology and human intervention on organization of the *C. baccatum* genepool. The results will facilitate utilization of *C. baccatum* for crop improvement and species conservation by providing a framework for efficient germplasm collection management and guidance for future plant acquisitions.

## Background

*Capsicum baccatum*, also known as the ají or Peruvian hot pepper, is a unique *Capsicum* species with origin in South America [1]. The species is divided into two major groups, the wild *C. baccatum* L. var. *baccatum* (formerly *C. microcarpum* Cav.) and the domesticated *C. baccatum* var. *pendulum* (Willd.) Eshbaugh. Morphological overlap occurs between the two forms of the species [2,3]. The wild form of the species typically bears small, erect, deciduous fruit, whereas the domesticated form typically bears larger sized, pendant and persistent fruit [4]. Human selection pressure promoted increased fruit size and weight with resultant downwards bending of the pedicel. Hence, the name ‘pendulum’ derived from the Latin pendulus for hanging fruit.

The earliest historical specimens of *C. baccatum* date to 2000 BC [5]. Historical documents demonstrate the significance of the fruit in ancient American, including Incan and earlier cultures. Contrary to the other four recognized domesticated *Capsicum* species (*C. annuum*, *C. chinense* Jacq., *C. frutescens* L. and *C. pubescens*), domesticated forms of *C. baccatum* are not commonly distributed outside South America. *C. baccatum* var. *pendulum* is the domesticated pepper of choice in Bolivia, Ecuador, Peru and Chile, and the most frequently grown pepper species in South America [6].

The center of origin for *C. baccatum* is believed to lie in Bolivia and southern Peru [4,7,8]. Domestication of the species is a relatively recent event in comparison to many cultivated crops and occurred approximately 4,500 years ago in Peru [3,9,10] and possibly at other sites of the present wild distribution range. Recent studies evaluating genus-specific starch fossils indicate the use of domesticated peppers as early as 6,000 years ago [11]. Utilizing amplified fragment length polymorphism (AFLP) genotypes [12], we previously demonstrated that genetic diversity in the wild form of *C. baccatum* (*C. baccatum* var. *baccatum*) was greater than in the domesticated form of the species (*C. baccatum* var. *pendulum*). Furthermore, we demonstrated admixture/shared ancestry between wild and domesticated *C. baccatum* botanical varieties and that the domesticated *C. baccatum* germplasm constitutes two principal genetic groups, largely based on their geographic distribution. One group was composed predominantly of cultigens from the western territories of the species’ distribution (Peru, Colombia, Ecuador, Bolivia, Chile and western Argentina) and the second of cultigens from the eastern regions (Brazil, Paraguay and eastern Argentina). The two genetic groups overlapped in the geographic location of present day Bolivia. The grouping pattern suggested that the cultigens of *C. baccatum* were domesticated at multiple sites and that their evolution followed two major lineages followed by lineage differentation. The wild accessions most closely related to the cultigens were found in the highlands of Peru and Bolivia, which supports the early hypothesis that this region is one of the domestication sites of this species. A Bayesian assignment analysis demonstrated that Brazilian wild forms of *C. baccatum* were genetically distant to all other accessions and made little to no contribution to the domesticated gene pool [12].

The United States Department of Agriculture, Agriculture Research Service (USDA, ARS) *C. baccatum* germplasm collection is comprised of accessions collected from a wide range of ecogeographical areas in South America. Phenotypic and genetic diversity of *Capsicum* in each of these areas is affected by geography, climate, ecology and human intervention. Improved understanding of the combined effects of these factors on the current structure of genetic diversity and morphological variation within the species is important for efficient germplasm conservation and use [13]. In the present study, a meta-analysis was utilized to describe diversity of the USDA, ARS *C. baccatum* germplasm collection using data from a geographic information system (GIS), morphological traits and AFLP-derived DNA fingerprints. Our objectives included: 1) mapping the ecogeographic distribution of the *C. baccatum* collection; 2) analyzing the spatial genetic structure in domesticated *C. baccatum* germplasm; and 3) assessing the relationship between the spatial genetic pattern and variation in morphological traits. The results will improve our knowledge of the structure of genetic diversity in *C. baccatum* germplasm, thus enhancing the conservation of this species and its utilization in pepper breeding.

## Results

### Ecogeographical distribution

**Figure 1:**
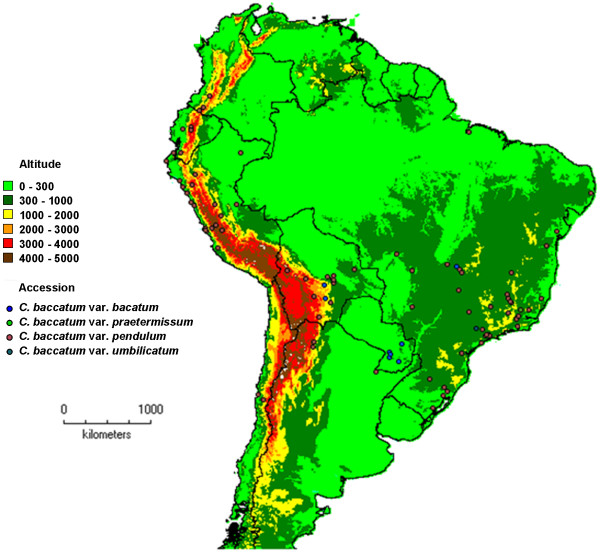
**Geographic distribution of the USDA, ARS *Capsicum baccatum *germplasm collection.** DIVA-GIS [14] was utilized for constructing the distribution map of the *C. baccatum* collection.

*Capsicum baccatum* occupies a diverse geographic range in South America (Figure 1, Tables 1 and 2). The maximal distance between any two accessions of the wild form of the species, *C. baccatum* var. *baccatum* and *C. baccatum* var. *praetermissum,* evaluated in this analysis was greater than 3,200 km and occurred between an accession from the Peruvian Andes, PI215699, and one from the coastal region in Southern Brazil, PI260533. The maximal distance between any two accessions of the domesticated form of the species, *C. baccatum* var. *pendulum* and *C. baccatum* var. *umbilicatum,* was greater than 5,000 km and occurred between an accession on the western coast of Peru, PI257151, and one on the eastern coast of Brazil, PI1520.

**Table 1:** **List of selected *Capsicum baccatum *accessions with GIS data from the USDA, ARS *Capsicum *germplasm collection**

**Source**	**Region**^**z**^	** Number of accessions**
*C. baccatum* var. *pendulum*		
Argentina	Corrientes, Jujuly, NA	3
Bolivia	Chuquisaca, Cochabamba, El Beni, La Paz, Potosí, Santa Cruz, Tarija, NA	26
Brazil	Bahia, Espírito Santo, Goiás, Mato Grosso, Mato Grosso do Sul, Minas Gerais, Pará, Paraná, Pernambuco, Rio de Janeiro, Rio Grande do Sul, São Paulo, NA	81
Chile	Cochimbo, NA	3
Colombia	Cauca, Nariño	2
Ecuador	Carchi, Chimborazo, Cotopaxi, El Oro, Imbabura, Loja, Los Rios, Tungurahura, NA	15
Paraguay	Alto Paraná, Asunción, Cordillera, Guaíra, Itapúa, Paraguarí	15
Peru	Amazonas, Ancash, Cajamarca, Huánuco, Ica, Junín, La Libertad, Lambayeque, Lima, Loreto, Pasco, Piura, Tumbes, Ucayali	52
*C. baccatum* var. *umbilicatum*		
Brazil	São Paulo	1
*C. baccatum* var. *baccatum*		
Bolivia	Chuquisaca, Cochabamba, Santa Cruz, NA	7
Brazil	Goiás, Rio Grande do Sul, NA	4
Paraguay	Caazapá, Cordillera, Paraguarí	5
Peru	Junín, NA	4
*C. baccatum* var. *praetermissum*		
Brazil	Minas Gerais	2

^z^NA, geographic data not available for all accessions.

**Table 2:** **Variation range of ecogeographic factors in the USDA, ARS *Capsicum baccatum *germplasm collection**

**Ecogeographic factors**	**Lower extreme**	**Upper extreme**
Latitudinal distance	−31	2.5
Longitudinal distance	−81.3	−35.3
Altitude (m.a.s.l)	1	4,008
Average annual temperatures (°C )	5	27
Maximum temperature (°C )	13	34
Minimum temperature (°C )	−5	22
Annual rainfall (mm)	3	2,849
Precipitation during the warmest quarter (mm)	0	1,226

The domesticated accessions (*C. baccatum* var. *pendulum, C. baccatum* var. *umbilicatum*) analyzed in this study covered a latitudinal distance ranging from −31 to 2.5 degrees and a longitudinal distance ranging from −81.3 to −35.3 degrees with a median of −17.8 degrees latitude/-57.2 degrees longitude in western Brazil, MatoGrosso do Sul, at the border of Bolivia (Figure 1). Accessions of the wild forms of the species (*C. baccatum* var. *baccatum*, *C. baccatum* var. *praetermissum*) covered a smaller range from −29.6 to −11.53 degrees latitude and −75.9 to −2.9 degrees longitude with a median of −20.8 degrees latitude /-57.1 degrees longitude in western Brazil, MatoGrosso do Sul, at the border of Bolivia and Paraguay.

*Capsicum baccatum* occupies altitudinal extremes across its geographic distribution (Figure 1, Table 2). Accessions, both wild and domesticated, grow from sea level (e.g. Pará, Brazil) to over 4,000 m (e.g. Junín, Peru). Approximately 50% of the accessions analyzed were found at altitudes below 500 m. The lowland accessions occur in the Amazon basin and the coastal regions of Ecuador, Peru, and Chile in the west and Brazil in the east. The highland accessions (above 2,000 m altitude) inhabit eastern and western sides of the Andes in Ecuador, Peru and Bolivia. Wild accessions occupy higher altitudes compared to domesticated ones. Fifty percent of the wild accessions grow at altitudes ranging from 210 m to 2,030 m (median 740 m). Approximately 50% of the domesticated accessions are found at altitudes ranging from 170 m to 780 m (median 510 m).

Average annual temperatures in the native *C. baccatum* habitat span 5°C (e.g. in Junín, Peru) to 27°C (e.g. Pará, Brazil and Loreto, Peru), with a median of 20°C for both wild and domesticated forms of the species (Table 2). Nearly 50% of the accessions evaluated occupy areas with average annual temperatures that range from 19°C to 22°C. The average minimum temperatures recorded for *C. baccatum* habitats are 6°C to 11°C for the wild accessions (*C. baccatum* var. *baccatum*, *C. baccatum* var. *praetermissum*) and 9°C to 13°C for the domesticated accessions (*C. baccatum* var. *pendulum, C. baccatum* var. *umbilicatum*). Approximately 10% of wild and domesticated accessions are regularly exposed to chilling temperatures (0–5°C), and those at high altitudes in the Andes (Junín, Peru) may experience freezing temperatures (−5°C).The warmest regions of the *C. baccatum* habitat are typically the continental areas, e.g. the Amazon basin of Brazil, but also tropical coastal regions such as those in Ecuador which are exposed to warm temperatures in the summer. In most areas of the distribution, *C. baccatum* is exposed to a maximum temperature of 28°C to 31°C and to over 34°C in a few of the continental or tropical coastal regions. In contrast, temperatures hardly fall below 25°C at some of the tropical habitat sites in the *C. baccatum* distribution.

Annual rainfall conditions are diverse in the *C. baccatum* distribution range (Table 2). Populations in regions along the western coast of South America are exposed to little rainfall during the summer months. In regions closer to the equator, higher temperatures are accompanied by greater amounts of rainfall. The majority of accessions analyzed, both wild and domesticated, receive 500–1,500 mm rainfall per year (median = 1,250 mm). Fifteen percent of the *C. baccatum* accessions evaluated grow in desert-like territories with less than 30 mm rainfall per year, e.g. Peruvian coastal areas. In contrast, on the eastern side of the Andes in the continental, tropical area of Peru (Loreto), precipitation rates are the highest for the *C. baccatum* distribution range, reaching almost 3,000 mm.

Notable with respect to the amount of rainfall in occupied habitats is the difference between *C. baccatum* var. *baccatum* and *C. baccatum* var. *pendulum*. While *C. baccatum* var. *baccatum* accessions did not occur at sites with less than 500 mm rainfall per year (e.g. Cochabamba, Bolivia), domesticated accessions occupied habitat with just 3 mm rainfall per year (La Libertad, Peru). Some domesticated accessions occupied areas with 0 mm precipitation during the warmest quarter of the year. In contrast, wild accessions in the collection were restricted to areas with a minimum of 150 mm during the warmest quarter. For both wild and domesticated accessions, the median amount of precipitation was 390 mm.

### Association between ecogeographic factors and morphological traits

Longitude, altitude, annual rainfall, annual temperature, maximum temperature during the warmest month, and minimum temperature during the coldest month were all associated with morphological variation in the 190 accessions of *C. baccatum* (Table 3). Among these ecogeographic factors, annual rainfall, followed by maximum temperature in the warmest month, precipitation in the warmest quarter and minimum temperature during the coldest quarter, exhibited the strongest associations with morphological traits. However, no association was detected between latitude and morphological traits in *C. baccatum* germplasm*.*

**Table 3:** **Association between ecogeographic factors and morphological traits in the USDA, ARS *Capsicum baccatum *germplasm collection**

**Morphological traits (Dependent)**	**Ecogeographic factors (Label)**	**F value**
Stem number	Precipitation warmest quarter	8.53^**^
Plant height	Longitude	5.00^*^
Plant habit	Minimum temperature coldest month	5.23^*^
Locule number	Annual rainfall	10.37^**^
Immature fruit color	Minimum temperature coldest month	5.02^*^
Fruit set (low to high)	Annual rainfall	4.54^*^
Fruit position (pendant to erect)	Maximum temperature warmest month	4.38^*^
Fruit persistance (seperating/persitent)	Longitude	6.23^*^
Days to maturity	Maximum temperature warmest month	9.06^**^
Days to maturity	Annual temperature	8.21^**^
Calyx shape (cup/saucer)	Precipitation warmest quarter	7.61^**^
Calyx shape (cup/saucer)	Altitude	6.50^*^
Calyx margin (smooth to serrate)	Annual rainfall	17.46^***^
Calyx margin (smooth to serrate)	Minimum temperature coldest month	6.94^**^
Calyx margin (smooth to serrate)	Annual temperature	6.12^*^
Anthocyanin immature fruit	Altitude	5.78^*^

^***, **, *^ Significant at 0.05, 0.01 and 0.001, respectively.

Substantial morphological differences were detected between the western and eastern groups (Table 4). For fruit attributes, fruit weight and fruit width in the western group were reduced relative to that recorded for the eastern group. Similarly, wall thickness was also reduced in the western group. No significant difference was detected for fruit length. Fruit maturation time was greater and fruit set slightly less for western accessions in comparison to eastern accessions. Immature fruits of eastern accessions are more likely to contain anthocyanin. The calyx margins are more serrate in varieties of the western group, and fruit persistence is somewhat reduced relative to eastern accessions.

**Table 4:** **Average morphological tratis in the ‘western’ and ‘eastern’ group of *Capsicum baccatum *germplasm accessions**

**Morphological traits**	**Western**	**Eastern**	***P*value**^**Z**^
Anther color [1 = yellow, 2 = yellow/blue]	1.08	1.03	NS
Anthocyanin immature fruit [1 = present, 0 = absent]	0.08	0.25	0.0313
Blossom end shape [1 = pointed, 9 = sunken]	1.88	2.14	NS
Calyx constriction [1 = present, 0 = absent]	0	0.02	NS
Calyx margin [1 = smooth, 9 = serrate]	5.05	3.87	0.0001
Calyx shape [1 = cup, 2 = saucer]	1.09	1.31	NS
Corolla color [1 = white, 2 = green-white, 5 = violet]	1 = 100%	1 = 100%	NS
Corolla spots [2 = yellow, 3 = green/yellow]	2 = 92%, 3 = 8%	2 = 89%, 3 = 11%	NS
Days to maturity [1 = early, 9 = late]	6.12	5.28	0.0142
Degree of pungency [1 = sweet, 9 = pungent]	5.68	5.71	NS
Determinate habit [1 = at least 75% mature at once, 0 = absent]	0.04	0.05	NS
Fasiculate [1 = present, 0 = absent]	0.05	0.12	NS
Fruit corkiness [0 = none, 9 = very high]	0.10	0.01	NS
Fruit length [cm]	6.00	5.80	NS
Fruit neck constriction [1 = present, 0 = absent]	0.02	0.08	NS
Fruit persistance [0 = separating calyx (has *Sp* gene), 0 = persistant calyx]	0.47	0.85	<0.001
Fruit pods per node	1.00	1.02	NS
Fruit position [1 = pendant, 9 = erect]	2.87	1.94	NS
Fruit set [1 = low, 9 = high]	6.25	6.83	0.0028
Fruit shape [1 = elongate, 2 = oblate, 4 = conic, 6 = bell]	1 = 88%, 4 = 12%,	1 = 81%, 2 = 2%, 4 = 9%, 6 = 9%	NS
Fruit weight [g]	4.40	6.00	0.014
Fruit width [cm]	1.70	2.00	0.0381
Immature fruit color [2 = green, 3 = green/yellow, 4 = yellow, 5 = white]	2 = 62%, 3 = 30%, 4 = 6%, 5 = 3%	2 = 52%, 3 = 40%, 4 = 3%, 5 = 5%	NS
Leaf pubescence [0 = glabrous, 9 = excessive]	0.05	0.02	NS
Leaf texture [1 = smooth, 9 = curled]	1.08	1.06	NS
Locule number	2.94	2.61	0.0063
Mature fruit color: Griffin + USDA observations [2 = red, 3 = orange-red, 4 = yellow, 5 = orange]	2 = 73%, 3 = 9%, 4 = 17%	2 = 89%, 3 = 3%, 4 = 1%, 5 = 7%	0.0536
Node anthocyanin [1 = present, 0 = absent]	0.89	0.92	0.0078
Pedicel position at anthesis [1 = pendant, 9 = erect]	8.36	8.79	<0.001
Peduncle insertion [1 = protruded, 9 = inserted]	2.69	3.12	NS
Peduncle length [cm]	4.40	3.90	NS
Plant habit [1 = sprawling, 9 = tall or upright]	5.54	5.18	NS
Plant height [cm]	61.70	74.70	<0.0011
Plant width [cm]	78.76	86.38	NS
Stem anthocyanin [1 = present, 0 = absent]	0.79	0.77	<0.001
Stem color [1 = green, 2 = purple]	1.00	1.00	NS
Stem number	6.52	5.73	0.0402
Stem pubescence [0 = glabrous, 9 = excessive]	0.85	0.69	NS
Stigma exsertion [1 = inserted, 9 = exserted]	4.44	4.13	NA
Wall thickness [cm]	0.14	0.19	0.0028

^Z^*P* value showing the differences between the western and eastern groups of *Capsicum baccatum*; NS, not significant; NA, not available.

With several exceptions, morphological differences for traits affecting plant architecture did not differ between western and eastern groups. The average plant height was 13 cm less in the west compared to the east, whereas stem number was greater.

### Morphological variation in wild and domesticated *C. baccatum*

Significant differences were observed in morphological traits between wild and domesticated *C. baccatum* accessions. Stem number, fruit length, width, weight, locule number, wall thickness, and peduncle length were all significantly reduced in *C. baccatum* var. *baccatum* versus *C. baccatum* var. *pendulum* (Table 5). All wild accessions exhibited abscising fruit while fruit of domesticated types were generally persistent. Corolla color of wild accessions was generally white or green-white and less frequently with violet coloration. Corollas of domesticated accessions lacked anthocyanin pigmentation. Similar to corolla pigmentation, domesticated accessions were less likely to exhibit anthocyanin pigmented immature fruit.

**Table 5:** **Average morphological traits in 190 accessions of wild and domesticated *Capsicum baccatum *germplasm accessions**

**Morphological traits**	**Wild**	**Domesticated**	***P*value**^**Z**^
Anther color [1 = yellow, 2 = yellow/blue]	0	0.06	NS
Anthocyanin immature fruit [1 = present, 0 = absent]	0.56	0.16	0.031
Blossom end shape [1 = pointed, 9 = sunken]	2.40	2.00	NS
Calyx constriction [1 = present, 0 = absent]	0	0.01	NS
Calyx margin [1 = smooth, 9 = serrate]	5.00	4.50	NS
Calyx shape [1 = cup, 2 = saucer]	1.00	1.21	NS
Corolla color [1 = white, 2 = green-white, 5 = violet]	1 = 77%, 5 = 23%	1 = 99%, 2 = 1%	4.26E-09
Corolla spots [2 = yellow, 3 = green/yellow]	2 = 100%	2 = 90%, 3 = 10%	NS
Days to maturity [1 = early, 9 = late]	6.60	5.70	0.022
Degree of pungency [1 = sweet, 9 = pungent]	6.21	5.70	NS
Determinate habit [1 = at least 75% mature at once, 0 = absent]	0	0.04	NS
Fasiculate [1 = present, 0 = absent]	0	0.09	NS
Fruit corkiness [0 = none, 9 = very high]	0.04	0.06	NS
Fruit length [cm]	3.64	6.13	<0.001
Fruit neck constriction [1 = present, 0 = absent]	0	0.05	NS
Fruit persistance [0 = separating calyx (has *Sp* gene), 0 = persistant calyx]	0	0.73	0.002
Fruit pods per node	1.00	1.01	NS
Fruit position [1 = pendant, 9 = erect]	5.93	1.96	NS
Fruit set [1 = low, 9 = high]	6.52	6.46	0.014
Fruit shape [1 = elongate, 2 = oblate, 4 = conic, 6 = bell]	1 = 71%, 2 = 14%, 4 = 14%	1 = 85%, 2 = 1%, 4 = 10%, 6 = 4%	NS
Fruit weight [g]	2.20	5.40	<0.001
Fruit width [cm]	1.22	1.92	0.001
Immature fruit color [2 = green, 3 = green/yellow, 4 = yellow, 5 = white]	2 = 75%, 3 = 25%	2 = 58%, 3 = 33%, 4 = 5%, 5 = 4%	NS
Leaf pubescence [0 = glabrous, 9 = excessive]	0.30	0.04	NS
Leaf texture [1 = smooth, 9 = curled]	1.09	1.08	NS
Locule number	2.78E + 00	2.80E + 00	0.027
Mature fruit color [2 = red, 3 = orange-red, 4 = yellow, 5 = orange]	2 = 70%, 3 = 10%, 5 = 20%	2 = 82%, 3 = 6%, 4 = 1%, 5 = 12%	NS
Node anthocyanin [1 = present, 0 = absent]	1.00	0.90	NS
Pedicel position at anthesis [1 = pendant, 9 = erect]	8.57	8.59	NS
Peduncle insertion [1 = protruded, 9 = inserted]	2.74	2.92	NS
Peduncle length [cm]	3.25	4.24	<0.001
Plant habit [1 = sprawling, 9 = tall or upright]	5.24	5.45	NS
Plant height [cm]	70.25	66.15	NS
Plant width [cm]	88.15	82.83	NS
Stem anthocyanin [1 = present, 0 = absent]	0.92	0.77	NS
Stem color [1 = present, 2 = purple]	1.00	1	NS
Stem number	5.95	6.01	0.022
Stem pubescence [0 = glabrous, 9 = excessive]	1.05	0.78	NS
Stigma exsertion [1 = inserted, 9 = exserted]	5.04	4.24	NA
Wall thickness [cm]	1.17E-01	1.68E-01	0.016

^Z^*P*-value showing the differences between the wild and domesticated *Capsicum baccatum* groups*;* NS, not significant; NA, not available.

**Figure 2:**
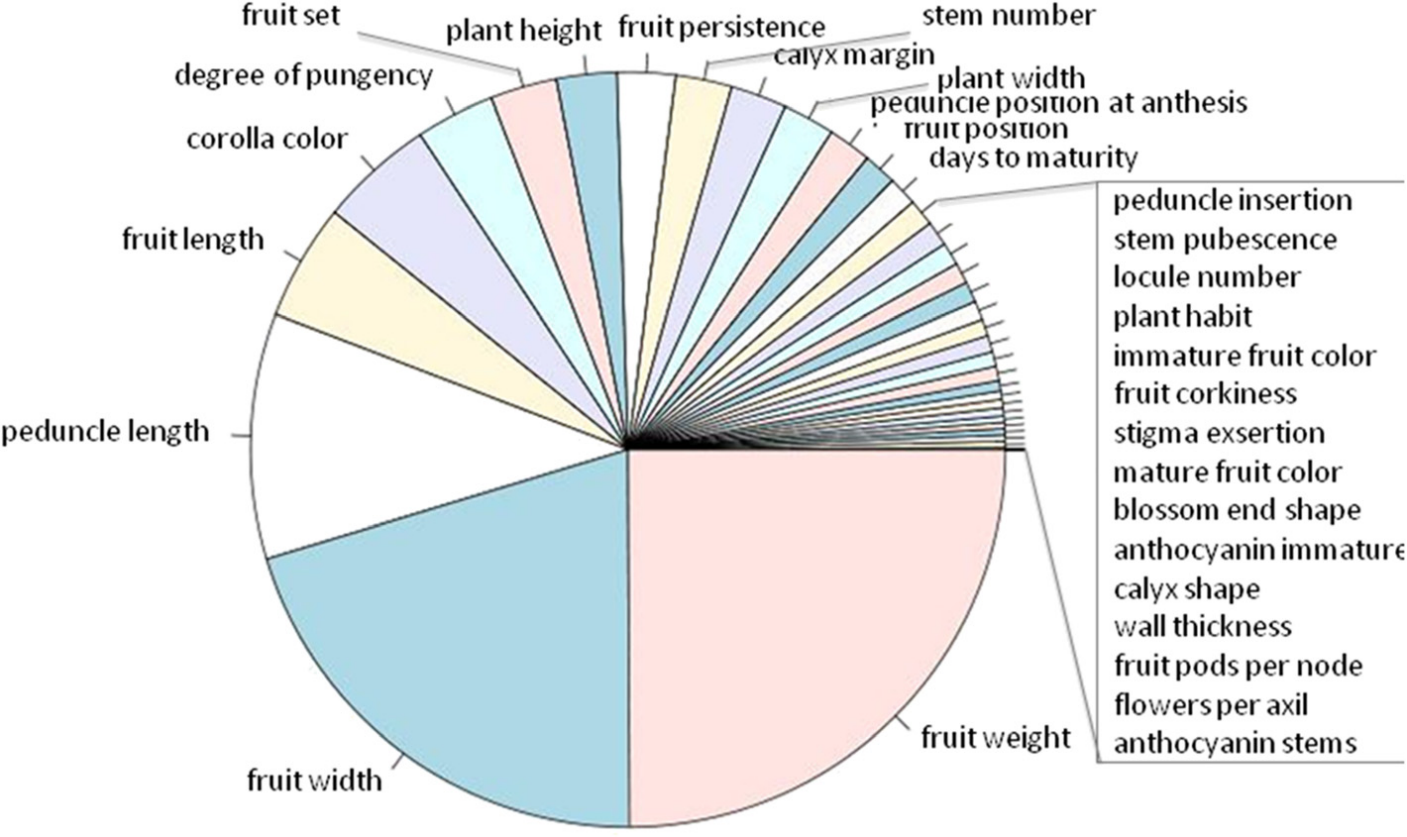
**Proportional importance of 40 morphological traits explaining the variation between wild and domesticated accessions.** The proportional importance was partitioned by Random Forest regression analysis (RandomForest, ver. 3.1).

Based on random forest estimates, 49% of the total morphological variation in *C. baccatum* can be explained by separation of the *C. baccatum* collection into two major groups, the wild and the domesticated forms of the species (Figure 2). Fruit weight was the variable of greatest importance (increasing node purity = 16.79) that distinguished wild versus domesticated types, followed by fruit width (increasing node purity = 13.22) and peduncle length (increasing node purity = 7.72). The contribution of features with the highest importance that distinguished the two taxa based on random forest regression (i.e. fruit weight, fruit width and peduncle length) was tested via multivariate analysis of variance (MANOVA). All four significance tests (Wilks, Hotelling-Lawley, Roy, Pillai) rejected the null hypothesis that the mean of the composite variable was the same as that for the individual groups (*C. baccatum* var. *baccatum* and *C. baccatum* var. *pendulum*), hence confirming the significance of the features for separation of the taxa. The proportion of the variance that is accounted for by the (composite) predictors was 28% (1 - Wilks’ lambda)*100 = (1 – 0.70)*100).

**Figure 3:**
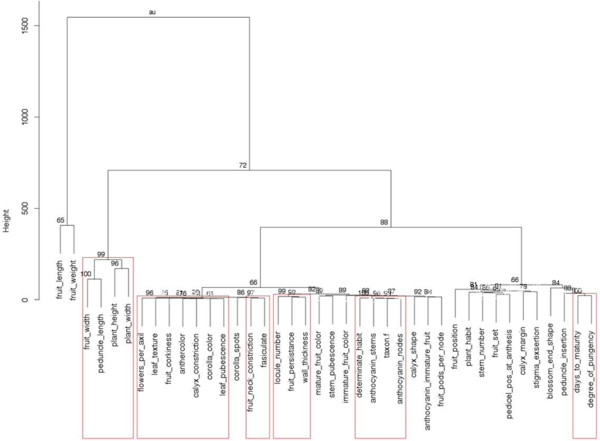
**Dendrogram depicting the relationship among 39 morphological traits in 177 accessions of *Capsicum baccatum *.** Distance measure of Minkowski [15] with 1,000 bootstrapping was used. AU values (%) are shown.

Cluster analysis demonstrated that *C. baccatum* fruit traits including fruit weight and fruit length are closely associated with each other and somewhat independent from the remainder of the morphological traits (Figure 3). Fruit width and peduncle length clustered together with plant height and plant width. Degree of pungency was associated with days to maturity. Anthocyanin production in vegetative plant parts, i.e. nodes and stems, was correlated.

### Spatial genetic structure

Mantel test revealed significant spatial correlation among the 174 accessions of domesticated *C. baccatum* (*r* = 0.17, *P* < 0.001). Correlation among the 92 accessions of the western group (*r* = 0.27, *P* < 0.001) was similar to that in the overall domesticated group. No significant correlation was detected among the 90 accessions of the eastern group (*r* = 0.05, *P* = 0.053).

**Figure 4:**
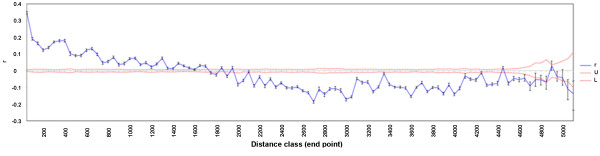
**Spatial autocorrelograms for 190 accessions of *Capsicum baccatum *var. *pendulum *showing the global spatial correlation (rc) as a function of geographical distance.** The 95% confidence interval about the null hypothesis of a random distribution was determined by bootstrapping. Even distance windows of 50 km were used with a limits of dispersal for *C. baccatum* var. *pendulum* at a distance of ca. 1,750 km.

Significant spatial genetic structure was detected in domesticated *C. baccatum* accessions at equally-sized distance, with distance class sizes of 50 km (Figure 4). The structure was maintained over geographic distances up to 1,750 km (= ‘limits of dispersal’). However, correlations were strongest (*r* = 0.2 to 0.35) within a radius smaller than 100 km.

**Figure 5:**
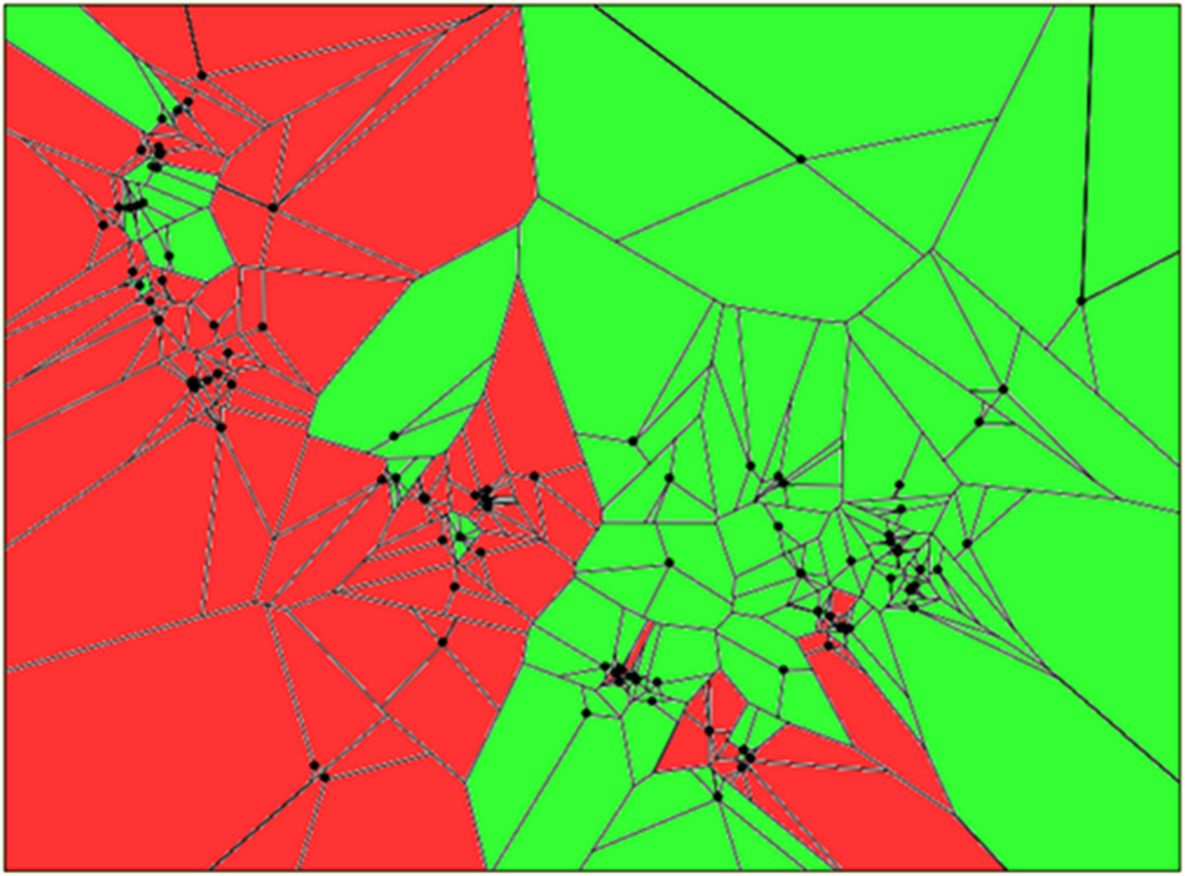
**Inferred clusters in the domesticated *Capsicum baccatum *accessions using a spatial population genetic analysis.** Analysis was based on the Bayesian clustering algorithm (Tess ver. 2.3.1; [16]). Points represent accessions and lines denote neighborhood networks based on Voronoi tessellation. Colors illustrate cluster membership. The parameter of burn in was set as 10,000 and the total number of sweeps was 50,000. Admixture was assumed and accounted for by the CAR model [17]. Runs were computed at K = 2 to K = 4. K = 3 is shown.

When a varying number of clusters were assumed, spatial population genetic analysis based on the Bayesian clustering algorithm demonstrated a division of the domesticated germplasm into two major groups, a western and an eastern spatial cluster (Figure 5). A small number of individuals shared membership among groups or geographic locations. Spatial cluster results provided a clearer delineation among western and eastern groups in comparison to our previous result based only on DNA fingerprints. The boundary between the two groups corresponded to today’s political borders for Peru/Bolivia and Paraguay/Brazil.

## Discussion

### Ecogographic distribution and adaptation

*Capsicum baccatum* displays a wide geographic distribution across the South American continent from the west coast to the east coast, and from Columbia in the north to Chile in the south. The variability of ecological and climatic conditions along the broad geographic range is extensive. This broad geographic range contributes to the great diversity found in *C. baccatum* and related cultivated and wild *Capsicum* genetic resources [18]. Wild and ancestral species of tomato, a related member of the Solanaceae, share an equally broad geographic distribution in South America from near sea level to over 3,300 m elevation, with habitat ranging from arid coastal lowlands to mesic uplands [19,20]. Analogous to *Capsicum* genetic diversity, the Andean geography, varied ecological habitats and different climates have contributed to wild tomato diversity [21]. Temperature extremes, as well as the amount and distribution of precipitation are often limiting factors to distribution of wild forms of a species and to a lesser extent cultivated forms. Ecological clines, i.e. associations between climatic conditions and a plant’s morphological or genetic patterns, arise as a consequence of migration and adaptations.

The ecological distribution of the *C. baccatum* accessions evaluated in this study indicated that domesticated accessions occupied habitat with rainfall as low as 3 mm per year, probably because of the option for irrigation. Wild forms of the species were restricted to areas of higher rainfall since cultivation is lacking. Likewise, cool temperatures in summer may represent a barrier to the distribution of wild forms of *C. baccatum*, as only domesticated types are found in areas where the maximum temperatures remain below 25°C. Genetic variation for pepper tolerance to moderately cold temperatures has been reported [22]. Assessment of five species including *C. annuum, C. frutescens, C. chinense, C. baccatum* and *C. pubescens,* revealed significant differences in low temperature (13°C – 18°C) seed emergence between accessions within species and a significantly greater seedling emergence score at low temperature for an accession of *C. baccatum* var. *pendulum* relative to all species accessions evaluated [22]. Similar to pepper, cold tolerance has been identified in wild as well as domesticated accessions of related Solanaceous crops that are native to temperate parts of the world where they may experience low temperatures [23,24]. For example, *Solanum lycopersicum* and *S. habrochaites* accessions native to Turkey and Peru, respectively, exhibited cold tolerance during seed germination as well as during vegetative growth [25].

Locally adapted populations of wild plant species typically differ in their responses to abiotic stresses, including extremes of moisture and temperature. Differences with respect to climatic factors between wild and domesticated forms of *C. baccatum* were not significant, possibly due to the low representation of wild accessions. Both wild and domesticated forms occur in areas with chilling or freezing temperatures; however, tolerance to low temperatures is likely more important for wild types which are perennial than for domestic forms that are grown seasonally. In climates with warmer temperatures, farmers had the opportunity to select for varieties with earlier flowering or shorterened fruit development time spanning the period from anthesis to fruit maturity. Despite the option for use of supplemental irrigation of cultivated forms, large fruit set in *C. baccatum* is only obtained in accessions collected from geographic areas with natural occurrence of high rainfall.

Apart from having its geographic center further west and north, the average annual temperature recorded for accessions in the western group is more than 1°C lower in comparison to accessions that comprise the eastern group. In addition, the annual rainfall as well as the precipitation amount during the warmest quarter is far less in the west in comparison to the east.

### Variation of morphological traits in the wild and domesticated *C. baccatum*

The present study revealed a high level of morphologic diversity, compatible with a prior report cataloguing aspects of *C. baccatum* morphological diversity [26]. Our results demonstrate morphological differences between the eastern and western groups that delineate the distribution range of wild and domesticated botanical forms of *C. baccatum*. Fruit attributes that contribute to yield potential, e.g. fruit weight, width and wall thickness, were generally greater in domesticated accessions occupying the eastern range in comparison to accessions from the western group. The differences in these domestication traits are likely the result of human selection and may reflect differences in cultural preferences between east and west.

Fruit traits in the domesticated *C. baccatum* accessions have been subjected to human selection [2,27,28]. Multiple factor analysis, MANOVA and random forest correlation identified fruit morphology as the most important phenotypic variable in *C. baccatum.* Out of the 40 morphological traits evaluated, we identified yield attributes that include fruit weight, fruit length and peduncle length as most informative for discrimination between wild (*C. baccatum* var. *baccatum*) and domesticated (*C. baccatum* var. *pendulum*) accessions. Fruit weight is clearly greater in domesticated accessions, as a result of enhanced fruit length, width and thicker fruit walls. Fruit width has greater importance than fruit length in the discrimination between wild and domesticated types; however, cluster analysis suggested that fruit length has a greater impact on final fruit weight. Studies in related Solanaceous crops suggest that a relatively small number of genes may account for the variation that discriminate wild and domesticated fruit of *C. baccatum.* In tomato, six loci explain much of the difference in fruit size evident between small fruited wild tomato species and their domesticated large fruited counterparts [29]. Orthologs of a number of these genes also account for size differences between small fruited ancestral eggplant species and large fruited commercial eggplant cultivars [30].

Differences in fruit morphology have been utilized to differentiate *C. baccatum* var. *baccatum* from *C baccatum* var. *pendulum*[7,31]. Subsequent analyses suggested that morphological differences between wild and domesticated types were not clear cut [2,3]. While we demonstrated that fruit yield-related attributes are robust indicators of varietal status, significant differences between varietal forms were also evident for traits including fruit anthocyanin pigmentation and fruit persistence. Related fruit attributes such as mature red pod color and upright peduncle orientation which are generally considered unique to wild varieties for bolstering bird predation and seed dissemination, did not distinguish wild from domesticated forms. For example, we found a number of domesticated varieties with small, erect fruit, and conversely, also wild types with larger, pendant fruit. A plausible explanation for this result may be the fact that *C. baccatum* is used as spice, and the use as a spice does not necessarily require substantial remodeling of fruit morphological traits such as increasing the fruit size, which would have led to a downward orientation of the peduncle. Our analyses and the descriptive results reported by [26] demonstrate introgresion of morphological characters among the botanical varieties. Wild and domesticated forms of the species are interfertile. Thus, hybridization likely accounts for much of this assimilation, particularly for geneflow from domesticated to wild accessions. Cultivation discourages geneflow from wild to domesticated genepools [12].

While most wild accessions bear fruit that produce anthocyanin when immature, that trait has largely been lost through human intervention during development of domesticated forms. Similar to anthocyanin in fruit, violet corollas are absent in the domesticated pool. Whereas fruit anthocyanin has been lost in domesticated accessions due to human selection, violet corollas are lacking in the lineage that contributed to domesticated forms of the species. Violet corollas are a trait that is exclusive to *C. baccatum* var. *praetermissum* wild types from Brazil. Our prior genetic analysis demonstrated that this wild form of *C. baccatum* is distinct and has not contributed to the domesticated pool [12]. Cluster analyses suggested an early divergence of the *C. baccatum* var. *praetermissum* lineage prior to *C. baccatum* domestication.

Cluster analysis of morphological traits demonstrated that anthocyanin pigmentation in vegetative plant parts (nodes, stems) is correlated but independent of anthocyanin accumulation in fruit. These observations are consistent with inheritance studies demonstrating simple inheritance for fruit anthocyanin pigmentation in *Capsicum* reproductive tissues and a complex inheritance for pigmentation in vegetative tissue [32].

Overall, the degree of fruit pungency was positively associated with days to maturity. The latter was inversely related to both maximum temperature and annual temperature. Although relationships between the degree of pungency and climatic factors were not significant, accessions with the highest pungency scores more often originated from warmer climates. Environmental factors, natural or brought upon by human intervention, such as temperature, light and fertilization level at the time of fruit maturation can influence fruit capsaicinoid concentration and pungency level and contribute to significant genotype x environement effects for this attribute [33-35]. Capsaicinoids are secondary metabolites that serve to deter predation by mammals with little effect on seed dispersal by birds since they do not sense capsaicinoid pungency.

Plant height is reduced and stem number increased among domesticated accessions in the ‘western’ group as based on both cluster [12] and Bayesian spatial analysis. Our data suggest that this is not a function of higher altitudes and cooler temperatures in the western territories. In fact, at lower temperatures, more erect plant habits are observed with thereby increased plant height, which may be a consequence of direct or indirect local human selection. A study of global patterns in plant height found that a wide range of height strategies were present in cold, dry, low productivity systems, but a lack of very short species in wetter, warmer, more productive sites [36]. That study found that the best model for global patterns in plant height included just one variable, precipitation in the wettest month. The longer maturation time and the lower fruit set observed for western *C. baccatum* accessions may be a consesquence of adaptations to climatic differences among the regions occupied by the western and eastern groups, i.e. based on lower temperatures and annual rainfall in western territories. Fruit set increased with annual rainfall, and highest fruit set was only observed in regions with at least 1,000 mm annual rainfall. In warmer habitats, maturation time or days to maturity were reduced. Alternatively or in addition to a scenario shaped by natural selection forces, human selection pressures for higher fruit set and shorter maturation times in the east may also have been stronger in this region in comparison to the west. Both annual rainfall and annual temperature are reduced in the western territories. In a study of two wild Andean tomato species, [37] proposed that local, regional, and species-wide environmental conditions are responsible for phenotypic and physiological diversification. Supportive of our results, Nakazato et al. [37] identified temperature and precipitation gradients as the strongest trait–environment associations, suggesting that those climatic factors are predominant drivers of adaptive diversification, at least in wild types. Due to assimilation of morphological attributes between wild and domesticated forms of *C. baccatum,* the relative role of natural selection versus human selection as drivers of morphological traits in the domesticated pool cannot be estimated from the present data.

### Spatial structure in domesticated *C. baccatum*

The domesticated C. *baccatum* germplasm was highly admixed and distributed across distant and ecologically diverse geographic regions. This is an indication that the domesticated *C*. *baccatum* remained well connected through gene flow. Over long distances, natural dispersal agents such as insects (pollen) and birds (seeds) likely play a less significant role for gene flow of domesticated material relative to human activities such as trade, because of much higher mobility based on the technical means of human transport [38]. A clear ‘immigrant’ identity was detected from the far end of the gene pool, i.e. among accessions that are separated by 3,000 km, demonstrating that human mediated, long-distance seed exchange occurred among distant regions. This result is in congruence with other domesticated crops such as beans [39] and maize [40] which exhibited significant seed-flow across long distances, in amounts and across distances where only transport by humans is possible. However, our results are contrary with a study of Mexican *C. annuum* populations [41], which suggested that human activities do not necessarily result in increased long distance gene flow relative to natural dispersal. The fact that ‘immigrants’ or introductions did not hybridize with local types indicates that: a) long-distance seed displacement occurred fairly recently; therefore, their genetic identity has not yet been obscured, or b) the specificities of the introduced types were maintained deliberately for their ethno-botanical purpose. Evidence for preservation of specific lineages within this *C. baccatum* germplasm was previously identified using AFLP markers for a group of accessions that form a distinct subclade nested within the predominantly Brazilian ‘eastern’ clade [12]. We called this group of Brazilian accessions the ‘umbilicatum’ clade as one of its member accessions was described as *C. baccatum* var. *umbilicatum*. This botanical variety was recently established [42]. This group exhibited greater divergence from the remainder of the accessions in the ‘eastern’ group, and was comprised of accessions from two areas of Brazil separated by over 1000 km. Conversely, other accessions in geographic proximity to ‘umbilicatum’ types were only distantly related to that subgroup. The role of ecological factors and agricultural selection in maintenance of landraces of pepper [43] and eggplant [44], Solanaceous relatives, have been reported.

Significant correlations were found between genetic and geographic distance with respect to the western and eastern subgroups of domesticated C. *baccatum*. Moreover, regional spatial structure was detected (within 100 km) in domesticated *C. baccatum*. The structure weakened after 100 km, although remained detectable up to almost 2,000 km. Adaptations to local ecological conditions may be responsible for regional differentiation. Our results are in congruence with a scenario of overlapping, short- to medium-distance trading units within the domesticated pool, which leave a signature of gradual decline in relatedness with increasing distance.

The observed spatial structure supports the conclusion that the distribution of *C. baccatum* var. *pendulum* genotypes is not random at the sampled geographical scale. Proximate genotypes tend to be more genetically similar than distant ones, consistent with the isolation by distance pattern of many other tropical species [37,45-47].

Multiple domestication events were proposed for the species’ based on the pattern of AFLP genetic admixture between wild and domesticated forms [12]. Our current results for spatial population analysis revealed separation of the western and eastern groups coincident with the political borders for Peru/Bolivia and Paraguay/Brazil. Wild accessions from areas that today comprise Bolivia and Peru were proposed as progenitors to the domesticated germplasm from these regions (the ‘western’ group), whereas Paraguayan wild types showed associations with the domesticated accessions from the same area [12]. The present results based on Bayesian spatial clustering methodology also demonstrate that each of the two sub-gene pools (the western and eastern) is homogeneous; indicating that the significant spatial genetic structure in each sub-gene pool is not due to recent colonization. The observed isolation by distance event in these two sub-gene pools therefore further support the hypothesis that the cultivated *C. baccatum* was domesticated independently in two sites, one in the Andes highlands (Peru/Bolivia) band and the other in the lowland of Paraguay.

## Conclusions

In summary, the present study mapped the ecogeographic distribution, analyzed the spatial genetic structure, and assessed the relationship between the spatial genetic pattern and the variation of morphological traits in a diverse *C. baccatum* germplasm collection spanning the species distribution range. We demonstrated that this species covers a great scope of ecogeographic diversity in South America, ranging from cool Andean highland to Amazonia rainforest. The high level of morphological diversity, with fruit weight as the leading variable, was revealed in a distribution pattern compatible to the AFLP-based two regional groups (western and eastern). The division of the domesticated germplasm into two major groups based on AFLP analysis was further supported by significant spatial structure. The results obtained further improve our understanding of the organization of the *C. baccatum* gene pool, thus enhancing the efficiency of conservation and utilization of this important *Capsicum* species.

## Methods

### Plant material

Seeds and passport data of *C. baccatum* accessions of all four known botanical varieties which includes the wild *C. baccatum* var. *baccatum* and *C. baccatum* var. *praetermissum*, and the domesticated *C. baccatum* var. *pendulum* and *C. baccatum* var. *umbilicatum*, from across the species’ entire distribution range, were obtained from the USDA, ARS *Capsicum* germplasm collection (USDA, ARS, Plant Genetic Resources Conservation Unit, Griffin, GA; Germplasm Resources Information Network [GRIN]; http://www.ars-grin.gov/) [48]. Selected accessions were representative of all countries that lie within the distribution range of the species and were chosen to maximize geographic distribution across the range. Wild *C. baccatum* germplasm is of limited availability in the *ex situ* collection, particularly for *C. baccatum* var. *baccatum* from Brazil (Jarret, personal communication). Passport data and accession IDs are reported in Albrecht et al. [12]. A total of 220 accessions were evaluated. Geographic information was available for 190 accessions and morphological data was available for 170 accessions. This included 20 accessions of *C. baccatum* var. *baccatum,* 197 *C. baccatum* var. *pendulum* accessions, two *C. baccatum* var. *praetermissum* and one *C. baccatum* var. *umbilicatum* accession (Table 1).

Five plants of each accession were grown in the field at the Beltsville Agricultural Research Center at Beltsville, Maryland using a completely randomized design [12]. Leaf tissue sampling, DNA extractions, AFLP fingerprinting and genetic diversity analysis (pairwise genetic distances derived from Jaccard genetic similarities) are described in [12]. Data comprising 40 morphological and physiological descriptors (see Table 4) for the *C. baccatum* germplasm collection was kindly provided by the USDA, ARS Plant Genetic Resources Conservation Unit and is available from GRIN. Climate data was obtained from DIVA GIS ( http://www.worldclim.org) [14].

### Ecogeographic distribution

Wild *C. baccatum* var. *baccatum, C. baccatum* var. *praetermissum* and the domesticated *C. baccatum* var. *pendulum* and *C. baccatum* var. *umbilicatum* accessions that comprise the germplasm collection were collected from a wide range of ecogeographic areas in South America with varied environments. To summarize the ecogeographic distribution of this collection, the average, range and variance of the ‘cornerstone’ climatic/geographic factors, i.e. those known to have the largest impact on plant physiology, including longitude, latitude, altitude, average annual temperature and rainfall, temperature extremes in the coldest/warmest month of the year and rainfall occurrence during the warmest season were computed using DIVA-GIS ver. 5.2.02 [14]. DIVA-GIS was also utilized for constructing the distribution map of the *C. baccatum* collection.

### Ecogeographical condition and morphological trait relationships

Logistic regressions were performed using the Proc REG procedure of SAS (SAS Institute Inc. 2008. SAS/STAT®9.2User’s Guide. Cary, NC: SAS Institute Inc.) to test for relationships between plant morphological traits and geographical locations and ecological conditions. Basic statistical descriptors and the test for differences between subgroups of *C. baccatum* derived from wild *C. baccatum* var. *baccatum* and *C. baccatum* var. *praetermissum* and the domesticated *C. baccatum* var. *pendulum* and *C. baccatum* var. *umbilicatum* were computed using R with the package ‘BSDA’ (ver. 2.11.1). Breiman’s Random Forest algorithm (RandomForest, ver. 3.1) was implemented for regressions among morphological and taxonomic features. Multivariate analysis of variance (MANOVA) was computed using R with the package ‘stats’. Dendrograms were computed with 10,000 bootstraps using R ‘pvclust’ with clustering measure ‘Ward’ and distance measure ‘Minkowski’ [15].

### Spatial genetic structure

Spatial genetic structure (SGS) in the *C. baccatum* germplasm collection was analyzed using a pairwise comparison of genetic similarity of individuals with respect to spatial distance separating those individuals, as implemented in GenAlEx 6 [49]. The significance of the autocorrelation coefficient (r) was tested by constructing a classic 2-tailed 95% confidence interval around the null hypothesis of no SGS (i.e., r = 0) and by performing 999 random permutations of genotypes among geographic locations [49]. In addition, a Mantel test was performed between the matrix of genetic distances and the linear pairwise geographical distances using the Mantel procedure in the same program. The test was first applied to the domesticated C. *baccatum* var. *pendulum* and *C. baccatum* var. *umbilicatum* accessions, followed by a separate test for the ‘western’ and the ‘eastern’ group, as defined by the Bayesian clustering analysis [12]. The Mantel test was also applied in smaller regions where accession representation was adequate, such as regions in Southern Peru and eastern Brazil. Spatial correlation was not tested for wild *C. baccatum* accessions due to small sample size.

A spatial population genetic analysis based on the Bayesian clustering algorithm (Tess ver. 2.3.1; [16]) was used to assign the domesticated *C. baccatum* accessions to geographical clusters. The parameter of burn in was set as 10,000 and the total number of sweeps was 50,000. Admixture was assumed and accounted for by the conditional autoregressive model (CAR) model [17]. Runs were computed for k = 2 to k = 4. For these analyses, only accessions with robust geographic location information were utilized.

## Abbreviations

AFLP: Amplified fragment length polymorphism; GIS: Geographic information system; CAR: Conditional autoregressive model; MANOVA: Multivariate analysis of variance; SGS: Spatial genetic structure; USDA, ARS: United States Department of Agriculture, Agricultural Research Service.

## Competing interests

Two of the authors (EA and ADM) are employed by Keygene which owns the AFLP trademark.

## Authors’ contributions

EA and JRS conceived and designed the study. EA, DZ, RAS and JRS evaluated morphological and geographic data. EA performed genotyping. EA, DZ and ADM conducted statistical analyses. EA, DZ and JRS wrote the manuscript. All authors reviewed the results from the data analysis and approved the final manuscript.
